# A new approach: preventive protocols with yeast products and essential oils can reduce the in-feed use of antibiotics in growing-finishing pigs

**DOI:** 10.1093/tas/txae104

**Published:** 2024-07-13

**Authors:** Ygor Henrique de Paula, Maíra Resende, Rhuan Filipe Chaves, Jéssica Aparecida Barbosa, Cesar Augusto Pospissil Garbossa, Matheus de Oliveira Costa, Fernanda Rigo, Robson Sfaciotti Barducci, Anderson Aparecido Dias Santos, Leticia Graziele Pacheco, Thaila Cristina Putarov, Vinícius de Souza Cantarelli

**Affiliations:** Animal Science Department, Federal University of Lavras, Lavras, Brazil; Animal Science Department, Federal University of Lavras, Lavras, Brazil; Animal Science Department, Federal University of Lavras, Lavras, Brazil; Animal Science Department, Federal University of Lavras, Lavras, Brazil; Department of Animal Nutrition and Production, School of Veterinary Medicine and Animal Science, University of São Paulo, Pirassununga, Brazil; Western College of Veterinary Medicine, University of Saskatchewan, Saskatoon, Canada; Imunova Análises Biológicas, Curitiba, Brazil; Biorigin, São Paulo, Brazil; Biorigin, São Paulo, Brazil; Biorigin, São Paulo, Brazil; Biorigin, São Paulo, Brazil; Animal Science Department, Federal University of Lavras, Lavras, Brazil

**Keywords:** diarrhea, growth promoters, microbiome, prebiotics, swine

## Abstract

The objective of this study was to evaluate the effects of yeast products (**YP**) and essential oils (**EO**) in total or partial replacement to in-feed antibiotic protocols (growth promoter and prophylactic), both in recommended doses and in overdose of prophylactic antibiotics (**PA**), on growth performance, and diarrhea incidence in the growing-finishing pigs; and fecal microbiota in market hogs. Four hundred pigs (20.36 ± 2.64 kg) were assigned to five treatments in a randomized block design: diets with prophylactic and growth promoter antibiotics (**ANT**); ANT with 30% more PA (**ANT+30**); diets with less PA and YP (**ANT+Y**); diets with less PA, YP and EO (**ANT+Y+EO**); and antibiotics-free diets with YP and EO (**Y+EO**). The content of the active components of the YP was 60% purified β-1,3/1,6-glucans extracted from *Saccharomyces cerevisiae* yeast (Macrogard), 20% functional water-soluble MOS (HyperGen), and 18% MOS, extracted from *Saccharomyces cerevisiae* yeast (ActiveMOS). From 0 to 14 d, pigs of the ANT+30, ANT+Y, and ANT+Y+EO treatments showed a greater body weight (**BW**) and average daily gain (**ADG**) compared to pigs from the Y+EO group. From 14 to 35 d, pigs of ANT+30 and ANT+Y+EO treatments were heavier than Y+EO group. At 105 d, ANT pigs had a higher BW than the Y+EO group. For the entire period, ADG of ANT pigs was greater, and feed conversion ratio better than Y+EO pigs. From 0 to 35 d, pigs of the Y+EO treatment showed a higher diarrhea incidence compared to pigs of the other groups. From 49 to 70 d, ANT+Y and ANT+Y+EO treatments showed a lower diarrhea incidence than Y+EO group, which remained the case during the overall period. At 105 d, the alpha diversity of fecal microbiota by Shannon Entropy was lower in ANT, ANT+30, and Y+EO groups than observed for ANT+Y+EO group. The abundance of *Firmicutes* phylum and *Firmicutes/Bacteroidetes* ratio was higher in ANT than in ANT+Y+EO pigs. *Proteobacteria* phylum abundance in ANT+Y+EO was higher than ANT, ANT+Y, and Y+EO. *Peptostreptococcaceae* family abundance was higher in ANT, ANT+30, and ANT+Y groups than in ANT+Y+EO and Y+EO groups. ANT+Y+EO and Y+EO groups show a lower abundance of *SMB53* genus than ANT and ANT+30 groups. In conclusion, the use of YP and EO, in partial replacement to the in-feed antibiotic protocols, does not reduce the growth performance, can replace antibiotic growth promotors, and reduce the in-feed use of PA in growing-finishing pigs. The use of YP and EO, together with PA, increases the microbial diversity, despite having important genera for weight gain in less abundance. Overdose of PA does not improve growth performance and reduces microbial diversity, which does not characterize it as an efficient preventive protocol.

## Teaser

The use of yeast products and essential oils as growth promoters and as prophylactic, in partial replacement to the in-feed antibiotic protocols, can replace antibiotic growth promotors, and reduce the use of prophylactic antibiotics in-feed in growing-finishing pigs.

## INTRODUCTION

In-feed antibiotics have been widely used to increase growth rates and prevent diseases in pigs (Van Boeckel et al., 2015), as much in the postweaning period as in the growing and finishing phases (Gaskins et al., 2002). However, routine use of antibiotics, and the overdose mainly, in food-producing animals has significantly contributed to the increasing emergence of multidrug resistant pathogens, incurring a major health concern in both animals and humans (Landers et al., 2012; Tang et al., 2017). There is currently a great interest in the production of pigs without antibiotics (Liu et al., 2018), which has been a reality for the European Union since 2006 (Dewulf et al., 2022). Therefore, to avoid the negative effects of removing antibiotics from the diets of pigs, changes in management and nutritional strategies may be required (Kil and Stein, 2010), as well as the use of validated alternative additives (Heo et al., 2013; Liu et al., 2018).

Yeast products (**YP**) are proposed as alternatives to antibiotics in the livestock industry (Burdick Sanchez et al., 2021). Dietary supplementation of YP has been paid increasing attention for improving immune function and intestinal development in swine (Broadway et al., 2015; Xu et al., 2018; Zhaxi et al., 2020). β-glucan, a functional polysaccharide of d-glucose monomers linked by β-glycosidic bonds (Stier et al., 2014), can modulate the immune system and stimulate a cascade of pathways that enhance both innate and adaptive immune responses (Vannucci et al., 2013), besides promotion on intestinal function (Xiong et al., 2015). Mannan oligosaccharides (**MOS**) prevent the adhesion of pathogenic bacteria to intestinal epithelial cells by attachment to the mannose-binding proteins expressed on the bacterial fimbriae (Kogan and Kocher, 2007; Spring et al., 2015). In addition, MOS supplementation evidenced additional beneficial properties such as decreased incidence of diarrhea (Zhao et al., 2012; Valpotić et al., 2016; Song et al., 2019) and higher growth performance (Miguel et al., 2004; Agazzi et al., 2020).

Essential oils (**EO**) are promising blends as novel antibacterial agents that can be used in pig production. Their bioactive compounds derived have immune, antioxidative, and antimicrobial properties (Sharifi-Rad et al. 2017). EO enhance digestibility (Liu et al., 2012; Chitprasert and Sutaphanit, 2014), reduce the production of cells and molecules involved in the immune response (Brenes and Roura, 2010; Liu et al., 2013), and promote gut health by minimizing the effect of the pathogenic bacteria (Chitprasert and Sutaphanit, 2014; Zhang et al., 2020). The modulation of gut microbiota, resulting in improvement of the growth performance, is also observed in piglets supplemented with EO (Li et al., 2018; Omonijo et al., 2018), therefore is so studied as alternative to antibiotics (Gong et al., 2013; Zhang et al., 2020). Studies that associate YP with EO are not found, as well as protocols with different inclusions of antimicrobial additives. In addition, partial replacement of prophylactic antibiotics (**PA**) with additive protocols has not yet been demonstrated. We understand that there is a potential synergism in this association that can contribute beneficially to the growth performance of pigs and reduce the total use of antibiotics (growth promoter and prophylactic forms).

The involvement of gut microbiota in host metabolism and health is well accepted (Lynch and Pedersen, 2016), but some specific roles remain to be studied. The intestinal microbiota plays crucial functions in nutrient digestion and absorption (Backhed et al., 2015), the development of the host immune (Postler and Ghosh, 2017), the differentiation of intestinal epithelium (Sommer and Backhed, 2013), and the maintenance of intestinal mucosal barrier (Garrett et al., 2010). There is also a possible link between the intestinal microbiota and growth performance, mainly feed efficiency (**FE**) in pigs (Yang et al., 2016; McCormack et al., 2017; Xiao et al., 2017; Tan et al., 2018). Therefore, the association of performance tests with microbiome technology in market hogs treated with different preventive protocols can provide a solid contribution to the understanding of the microbiota and host metabolism relationship.

We hypothesized that replacing antibiotic growth promoters (**AGP**) with nutritional additives or reducing the frequency of use of PA, due to the inclusion of these additives, do not decrease the growth performance of pigs in the growing-finishing phase and modify the fecal microbiota in market hogs. Therefore, the objective of this study was to evaluate the effects of YP and EO in total or partial replacement to the in-feed antibiotic protocols (growth promoter and prophylactic), both in recommended doses and in overdose of PA, on growth performance, in the total consumption of antibiotics during the experimental period, diarrhea incidence in the growing-finishing pigs, and fecal microbiota in market hogs.

## MATERIALS AND METHODS

### Animals, Experimental Design, and Housing

The experimental design and procedures were approved by the Ethics Committee on Animal Use of University of São Paulo under Protocol number 2172090321. The experiment was conducted in the growing-finishing facilities of the Animalnutri Research Center located within a commercial pig farm in Patos de Minas, Brazil. A total of 200 barrows and 200 gilts (DanBred sows and LQ1250 sires) with an average initial body weight of 20.36 ± 2.64 kg (63 d of age) were randomly allocated in a randomized block design (sex and initial body weight were the blocking factor). The experiment had five treatments: diets with prophylactic and growth promoter antibiotics (**ANT**); ANT with 30% more prophylactics antibiotics (**ANT+30**); diets with less prophylactics antibiotics and YP as prophylactic and growth promoter (**ANT+Y**); diets with less prophylactics antibiotics, YP, and EO as prophylactic and growth promoter (**ANT+Y+EO**); and antibiotics-free diets with YP and EO as prophylactics and growth promoter (**Y+EO**). The treatments are further detailed in Table 1. Eight replicates (10 pigs/pen) were used in the trial. The PA were composed of 200 ppm tiamulin and 440 ppm amoxicillin, and the overdose was 30% higher PA. The AGP used was 10 and 5 ppm enramycin in growing and finishing phases, respectively. The content of the active components of the yeast products 1 was 60% purified β-1,3/1,6-glucans extracted from *Saccharomyces cerevisiae* yeast (Macrogard, Biorigin, São Paulo, Brazil). The content of the active components of the yeast products 2 was 20% functional water-soluble MOS (HyperGen, Biorigin, São Paulo, Brazil). The content of the active components of the yeast products 3 was 18% MOS, extracted from *Saccharomyces cerevisiae* yeast (ActiveMOS, Biorigin, São Paulo, Brazil). The contents of the active components of the EO were a blend of 12% carvacrol and 6% cinnamaldehyde, capsaicin, anethole, and cineole (Activo, GRASP, Curitiba, Brazil).

**Table 1.** Experimental treatments

Treatments	Growing phase				Finishing phase	
	0-14 d	14-35 d	35-49 d	49-70 d	70-84 d	84-105 d
ANT	PA^a^	AGP^b^	PA	AGP	PA	AGP
ANT+30	Overdose PA^c^	AGP	Overdose PA	AGP	Overdose PA	AGP
ANT+Y	PA + YP1^d^	YP2^e^	YP1 + YP2	YP3^f^	PA + YP1	YP3
ANT+Y+EO	PA + YP1	YP2 + 200 ppm EO	YP1 + YP2 + 400 ppm EO	YP3	PA + YP1	YP3 + 200 ppm EO
Y+EO	YP1 + YP2 + 400 ppm EO^g^	YP2 + 200 ppm EO	YP1 + YP2 + 400 ppm EO	YP3	YP1 + YP2 + 400 ppm EO	YP3 + 200 ppm EO

^a^Prophylactic antibiotics (PA): 200 ppm tiamulin and 440 ppm amoxicillin.

^b^Antibiotics growth promoter (AGP): 10 and 5 ppm enramycin in growing and finishing phases, respectively.

^c^30% higher prophylactic antibiotic: 260 ppm tiamulin and 572 ppm amoxicillin.

^d^YP1: 300 ppm of 60% purified β-1,3/1,6-glucans, from *Saccharomyces cerevisiae* yeast.

^e^YP2: 1,000 ppm of 20% functional water-soluble MOS.

^f^YP3: 1,500 ppm of 18% MOS, from *Saccharomyces cerevisiae* yeast.

^g^Blend of essential oils included 12% carvacrol and 6% cinnamaldehyde, capsaicin, anethole and cineole.

The animals were housed in pens allowed a floor space of 1.40 m² per pig and had a partially slatted floor. All piglets were provided with feed and water in a five-space feeder (semiautomatic) and nipple drinkers.

### Diets and Experimental Procedures

The experimental period was 105 d and it used six diets, all pigs were fed the same basal diet. The basal diet was formulated to meet or exceed the nutritional specifications suggested by Rostagno et al. (2017) for pigs during the growing to finishing phases (Supplementary Table S1) and the additives were added according to each treatment. The pigs had ad libitum access to feed and water throughout the experimental period. The basal diet did not contain growth promoter additives.

Feed intake per pen and individual body weights were recorded at 0, 14, 35, 49, 70, 84, and 105 d. Based on these data, the ADG, ADFI, feed conversion ratio (**FCR**), and the total consumption of antibiotics were calculated. For fecal scoring, the feces present in the pen were assessed daily and graded as normal feces (no diarrhea) or liquid or pasty stools (presence of diarrhea), following the method of Casey et al. (2007). At the end of the trial, the occurrence of diarrhea was calculated as percentage of the phase.

### Sample Collections

On day 105 of the trial, fecal samples were collected from the six average-weight replicates (pens), totaling 36 samples. The three pigs with the closest individual ADG to the group ADG mean were chosen. Fecal samples were collected by digital rectal stimulation and a pool with these three samples was formed. All fecal samples were immediately frozen in liquid nitrogen and stored at −80 °C until analyses.

### Fecal Microbiota Analyses

Total bacterial DNA was extracted from the fecal sample contents by using a commercial kit (ZR Fecal DNA MiniPrep kit; Zymo Research Corp., Irvine, CA, USA) according to the manufacturer’s instructions. The extracted DNA was quantified by spectrophotometry at 260 nm using a NanoDrop 2000 spectrophotometer (Thermo Fisher Scientific Inc., Wilmington, DE, USA). To assess the integrity of the extracted DNA, all samples underwent electrophoresis in 1% agarose gel, were stained with a 1% ethidium bromide solution, and visualized with ultraviolet light in a transilluminator.

Thereafter, the variable V4 region of the 16S rRNA gene was amplified using the universal primers 515F and 806R and KlenTaq Master Mix (Sigma). Amplification controls without a template were employed. The PCR conditions used were: 94 °C for 3 min (1 cycle), 94 °C for 45 s/50 °C for 30 s/68 °C for 60 s (18 cycles), and a last step of 72 °C for 10 min. The amplicons were quantified with Qubit using an HS dsDNA kit (Invitrogen), diluted to 500 pM, and pooled. Then, 16 pM of pooled DNA were sequenced using MiSeq reagent 500V2 (Degnan and Ochman, 2012). Sequencing was performed using an Illumina MiSeq sequencer (Illumina) obtaining paired-end reads of 250 bp as described.

For the sequences obtained, the data filtering was completed by removing low-quality base. The sequences after trimming were analyzed with the QIIME pipeline (Caporaso et al., 2010), including the extraction of operational taxonomic units (**OTUs**) and overlapping analyses of OTUs. OTUs were clustered with a 97% similarity threshold. To compare the sequences, the 2017 update (SILVA 128) of the SILVA ribosomal sequences database (Yilmaz et al., 2014) was used. To generate the classification of bacterial communities by identifying OTUs, 22,110 reads per sample were used. This was in order to normalize the data and not compare samples with a different number of reads, thus avoiding a sample bias.

### Nucleotide Sequence Accession Number

All the raw sequences obtained after assembling and filtering were submitted to the NCBI site under accession number BioSample: PRJNA721984.

### Statistical Analyses

The pen was used as the experimental unit for the statistical analysis of growth performance and antibiotics consumption, and the pooled samples were used as the experimental unit for the microbiome analyses. The data were analyzed using the SAS software statistical package 9.3 (SAS, Cary, NC), except for sequencing data. The Shapiro–Wilk test was used to evaluate parametric data distribution. If the data did not have a normal distribution, data transformation was performed using PROC RANK. The effects were analyzed using the SAS MIXED procedure appropriate for a randomized block design (initial weight and sex). When the *F* test (*P* < 0.050) showed a significant difference, Tukey’s test was used to compare the means, with a significance level of 0.050. To analyze the diarrhea incidence, a generalized linear model (binomial analysis) was performed using the GENMOD procedure of SAS 9.3, with a significance level of 0.050. DNA sequencing was performed using the statistical metagenomics program STAMP: Statistical Analysis of Metagenomic Profiles. To compare the abundance of the genders identified between treatments, ANOVA (*P* < 0.050) with Tukey–Kramer post hoc test and Benjamini–Hochberg FDR multiple test correction was used. Only statistically different results were shown. The averages for biodiversity between treatments were compared using the number of OTUs and the Kruskal–Wallis test (*P* < 0.050), because they presented a nonparametric distribution according to the Shapiro–Wilk test. The *Firmicutes/Bacteroidetes* (**F/B**) ratio means between different groups was compared by one-way ANOVA (*P* < 0.050) with Tukey’s multiple comparisons test. The microbiome figures and their legends were automatically generated by the STAMP program.

## RESULTS

### Growth Performance

The growth performance results of the pigs during the experimental period are presented in Table 2. In the growing I phase (0 to 14 d), the pigs of the ANT+30, ANT+Y, and ANT+Y+EO treatments showed a greater BW (*P* = 0.001) and ADG (*P* = 0.001) compared to the pigs from the Y+EO group. The pigs of ANT+Y+EO group had better ADG than ANT pigs. In the growing II phase (14 to 35 d), the pigs of the ANT+30 and ANT+Y+EO treatments were heavier than Y+EO group (*P* = 0.010). In the growing III and IV, and finishing I phases, there were no differences between treatments for the performance variables. In the last phase (finishing II, 84 to 105 d), the ANT pigs had a higher BW than the Y+EO group (*P* = 0.023), but similar results to ANT+30, ANT+Y, and ANT+Y+EO treatments. In this phase, the pigs of ANT+30 and Y+EO groups had lower ADG than ANT pigs (*P* = 0.007). When taking into account the entire experimental period, the ADG of the ANT pigs was greater than Y+EO pigs but similar results to the ANT+30, ANT+Y, and ANT+Y+EO groups (*P* = 0.025). The pigs of ANT group had better FCR than Y+EO pigs and similar to other treatments (*P* = 0.011).

**Table 2.** Effects of experimental diets on growth performance in pigs from growing to finishing phase

Item^1^	Treatments^2^					SEM	*P*-value^3^
	ANT	ANT+30	ANT+Y	ANT+Y+EO	Y+EO		
Initial BW, kg	20.369	20.364	20.364	20.363	20.364	1.022	0.999
Growing I, days 0 to 14							
BW 14 d, kg	33.237^ab^	33.725^a^	33.711^a^	33.925^a^	32.850^b^	1.237	0.001
ADFI, kg	1.338^ab^	1.390^a^	1.393^a^	1.402^a^	1.323^b^	0.052	0.092
ADG, kg	0.919^bc^	0.954^ab^	0.953^ab^	0.968^a^	0.891^c^	0.020	0.001
FCR	1.458	1.456	1.456	1.445	1.484	0.035	0.672
Growing II, days 14 to 35							
BW 35 d, kg	52.125^ab^	53.050^a^	52.675^ab^	53.381^a^	50.409^b^	1.681	0.010
ADFI, kg	1.825	1.861	1.863	1.891	1.789	0.078	0.149
ADG, kg	0.900	0.920	0.903	0.926	0.868	0.028	0.266
FCR	2.028	2.020	2.062	2.040	2.017	0.032	0.469
Growing III, days 35 to 49							
BW 49 d, kg	67.813	68.925	67.613	68.581	66.211	1.985	0.143
ADFI, kg	2.494	2.535	2.426	2.450	2.361	0.151	0.248
ADG, kg	1.121	1.134	1.067	1.125	1.088	0.045	0.577
FCR	2.222	2.245	2.273	2.267	2.183	0.066	0.750
Growing IV, days 49 to 70							
BW 70 d, kg	91.520	91.441	90.648	91.472	89.692	2.405	0.446
ADFI, kg	3.109	3.051	3.043	3.068	3.020	0.226	0.691
ADG, kg	1.129	1.077	1.062	1.074	1.085	0.038	0.126
FCR	2.750	2.892	2.863	2.818	2.779	0.105	0.198
Finishing I, days 70 to 84							
BW 84 d, kg	108.840	108.580	108.120	108.090	106.570	2.586	0.410
ADFI, kg	3.477	3.398	3.574	3.501	3.526	0.184	0.189
ADG, kg	1.218	1.224	1.234	1.187	1.205	0.024	0.680
FCR	2.813	2.784	2.905	2.950	2.929	0.118	0.127
Finishing II, days 84 to 105							
BW 105 d, kg	131.430^a^	129.120^ab^	129.410^ab^	129.660^ab^	127.090^b^	2.485	0.023
ADFI, kg	3.504^a^	3.337^b^	3.372^ab^	3.423^ab^	3.427^ab^	0.126	0.048
ADG, kg	1.129^a^	1.026^b^	1.064^ab^	1.078^ab^	1.025^b^	0.020	0.007
FCR	3.108	3.221	3.177	3.181	3.311	0.133	0.109
Total phase, days 0 to 105							
ADFI, kg	2.654	2.614	2.629	2.650	2.601	0.133	0.472
ADG, kg	1.060^a^	1.042^ab^	1.039^ab^	1.047^ab^	1.021^b^	0.017	0.025
FCR	2.482^b^	2.505^ab^	2.528^ab^	2.527^ab^	2.563^a^	0.083	0.011

^1^BW, body weight; ADG, average daily gain; ADFI, average daily feed intake; FCR, feed conversion ratio; SEM, standard error of the mean.

^2^ANT: basal diet with antibiotics; ANT+30: basal diet with antibiotics in overdose; ANT+Y: basal diet with antibiotics and yeast products; ANT+Y+EO: basal diet with antibiotics, yeast products and essential oils; Y+EO: basal diet with yeast products and essential oils.

^3^Different lowercase letters indicate significant differences between groups according to Tukey’s test, *P* < 0.050. Data are expressed as means (8 replicates/treatment).

### Antibiotics Consumption

As shown in Table 3, the experimental treatments provided significant variations regarding the amount of antibiotic consumed in each of the groups during the evaluation of the entire period. The pigs of the ANT+30 treatment showed the highest consumption of tiamulin (*P* < 0.001), amoxicillin (*P* < 0.001), and total antibiotics (*P* < 0.001). The ANT treatment obtained a higher consumption of tiamulin, amoxicillin, and total antibiotics than the ANT+Y and ANT+Y+EO groups, and lower when compared to the ANT+30 treatment. There were no differences between the ANT+Y and ANT+Y+EO treatments for all variables. Pigs from ANT and ANT+30 treatments showed no difference in enramycin consumption. As it is an antibiotic-free treatment, the Y+EO group does not show antibiotic consumption values, as well as the ANT+Y and ANT+Y+EO groups when analyzing enramycin.

**Table 3.** Effects of experimental diets on antibiotics consumption (g) in pigs from growing to finishing phase

Item	Treatments^1^					SEM^2^	*P*-value^3^
	ANT	ANT+30	ANT+Y	ANT+Y+EO	Y+EO		
Tiamulin, g	20.466^b^	26.657^a^	13.908^c^	13.727^c^	0.00^d^	0.435	<0.001
Amoxicillin, g	45.024^b^	58.645^a^	30.598^c^	30.200^c^	0.00^d^	0.957	<0.001
Enramycin, g	1.404^a^	1.379^a^	0.00^b^	0.00^b^	0.00^b^	0.024	<0.001
Total antibiotics, g	66.894^b^	86.680^a^	44.506^c^	43.927^c^	0.00^d^	1.414	<0.001
Total antibiotics, %^4^	100.00	129.578	66.532	65.667	0.00	—	—

^1^ANT: basal diet with antibiotics; ANT+30: basal diet with antibiotics in overdose; ANT+Y: basal diet with antibiotics and yeast products; ANT+Y+EO: basal diet with antibiotics, yeast products and essential oils; Y+EO: basal diet with yeast products and essential oils.

^2^SEM, standard error of the mean.

^3^Different lowercase letters indicate significant differences between groups according to Tukey’s test, *P* < 0.050. Data are expressed as means (eight replicates/treatment).

^4^Relative amount of antibiotic use between groups with ANT treatment as a reference value of 100%.

### Diarrhea Incidence

**Figure 1.**
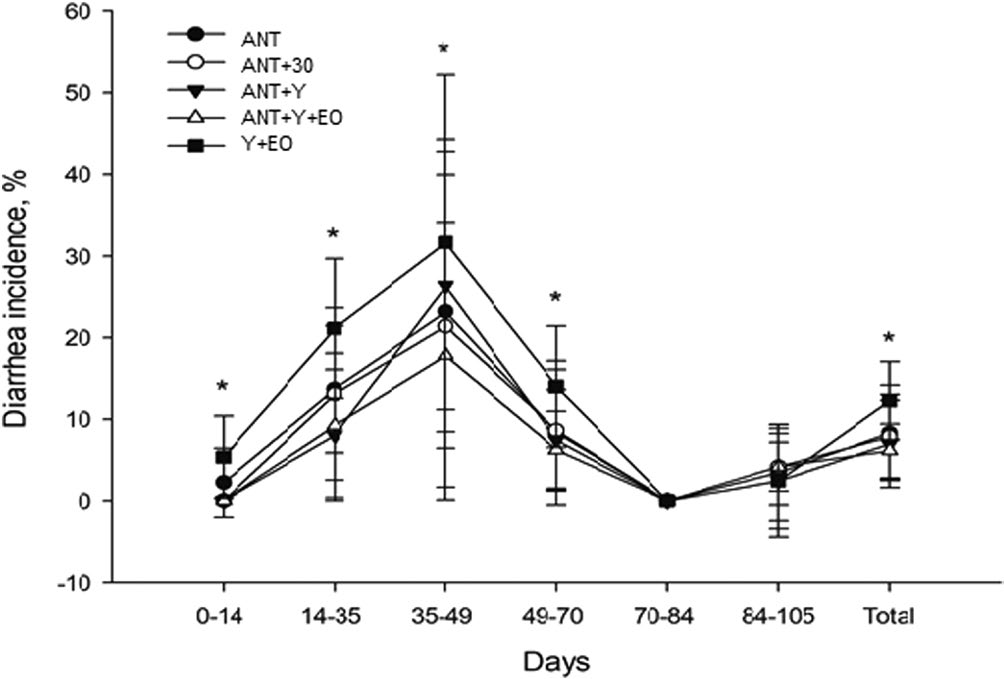
Effect of experimental diets on the diarrhea incidence in pigs from growing to finishing phase. ANT: basal diet with antibiotics; ANT+30: basal diet with antibiotics in overdose; ANT+Y: basal diet with antibiotics and yeast products; ANT+Y+EO: basal diet with antibiotics, yeast products, and essential oils; Y+EO: basal diet with yeast products and essential oils. Data are expressed as means (eight replicates/treatment) and SEM is represented by vertical bars. *Significant differences between groups according to binomial analysis, *P* < 0.050.

As shown in Fig. 1, during the growing phases I (0 to 14 d) and II (14 to 35 d), the pigs of the Y+EO treatment showed a higher diarrhea incidence compared to the pigs of the other groups (*P* < 0.001). In growing phase III (35 to 49 d), the ANT, ANT+30, and ANT+Y+EO treatments had a lower diarrhea incidence than the pigs of Y+EO group (*P* = 0.019). From 49 to 70 d (growing phase IV), the ANT+Y and ANT+Y+EO treatments showed a lower diarrhea incidence than the Y+EO group (*P* = 0.005), which remained the case during the overall period (*P* < 0.001). In the finishing I and II phases, there were no differences between treatments for diarrhea incidence. Considering the entire period, the ANT+Y+EO group had a lower diarrhea incidence than the ANT+Y group. The treatments had no effects on the diarrhea incidence from the 70th to 105th day of the trial (*P* = 0.631).

### Fecal Microbiota

**Figure 2.**
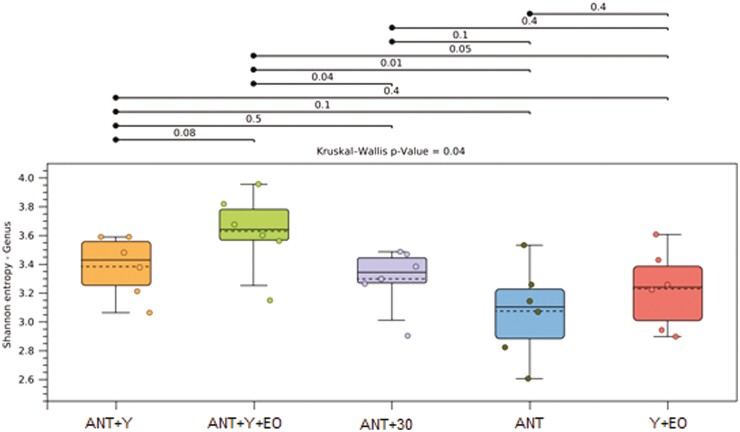
Effect of experimental diets on the alpha diversity by Shannon Entropy in market hogs. ANT+Y: basal diet with antibiotics and yeast products; ANT+Y+EO: basal diet with antibiotics, yeast products, and essential oils; ANT+30: basal diet with antibiotics in overdose; ANT: basal diet with antibiotics; Y+EO: basal diet with yeast products and essential oils. Piglet is an experimental unit; six piglets/treatment.

**Figure 3.**
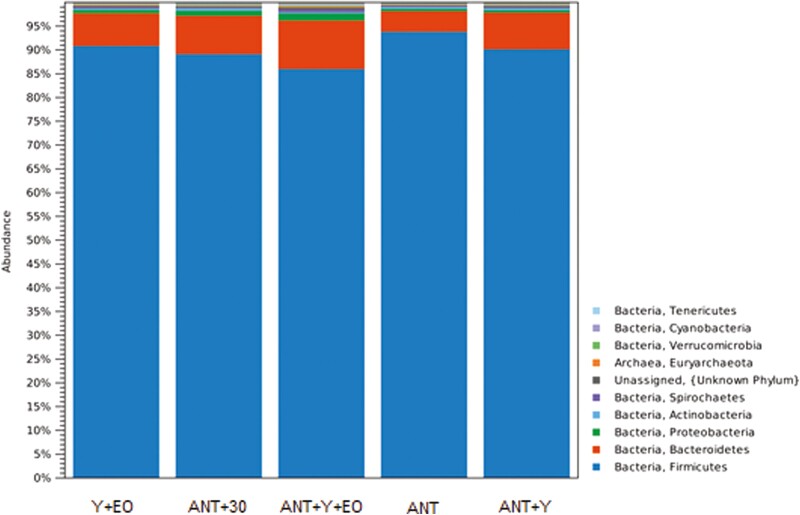
Effect of experimental diets on the phylum levels in market hogs. Y+EO: basal diet with yeast products and essential oils; ANT+30: basal diet with antibiotics in overdose; ANT+Y+EO: basal diet with antibiotics, yeast products, and essential oils; ANT: basal diet with antibiotics; ANT+Y: basal diet with antibiotics and yeast products. The phyla present differ significantly in their abundance between treatments according to the Kruskal–Wallis test (*P* < 0.050). Piglet is an experimental unit; six piglets/treatment.

**Figure 4.**
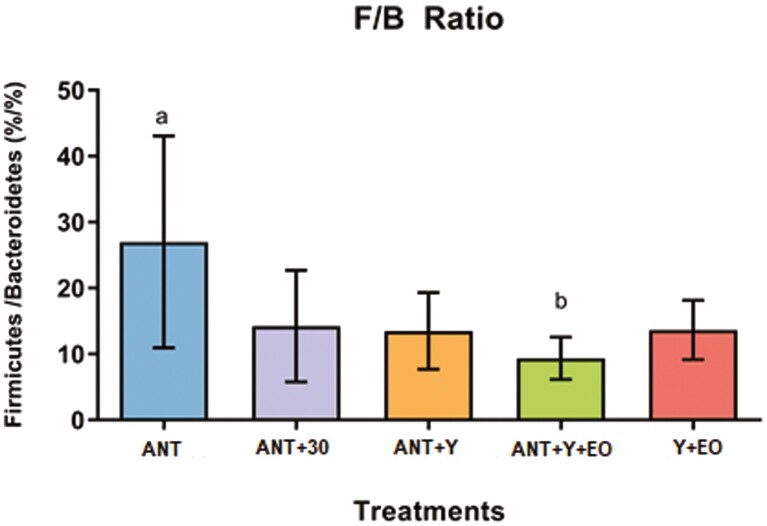
Effect of experimental diets on the *Firmicutes/Bacteroidetes* (F/B) ratio in market hogs. ANT: basal diet with antibiotics; ANT+30: basal diet with antibiotics in overdose; ANT+Y: basal diet with antibiotics and yeast products; ANT+Y+EO: basal diet with antibiotics, yeast products, and essential oils; Y+EO: basal diet with yeast products and essential oils. The F/B ratio means between different groups was compared by one-way ANOVA (*P* < 0.050) with Tukey’s multiple comparisons test. Piglet is an experimental unit; six piglets/treatment.

**Figure 5.**
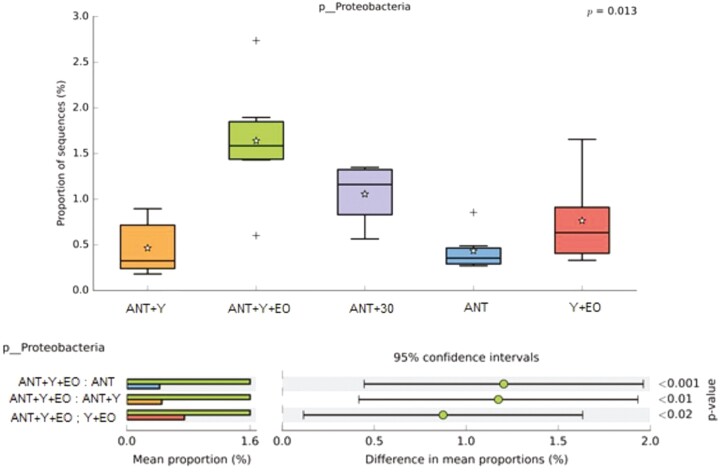
Effect of experimental diets on *Proteobacteria* phylum relative abundance in market hogs. (A) Box plot showing the distribution in the proportion of *Proteobacteria* assigned to samples from treatments. (B) Post hoc plot for *Proteobacteria* indicating. ANT+Y: basal diet with antibiotics and yeast products; ANT+Y+EO: basal diet with antibiotics, yeast products, and essential oils; ANT+30: basal diet with antibiotics in overdose; ANT: basal diet with antibiotics; Y+EO: basal diet with yeast products and essential oils. Piglet is an experimental unit; six piglets/treatment.

**Figure 6.**
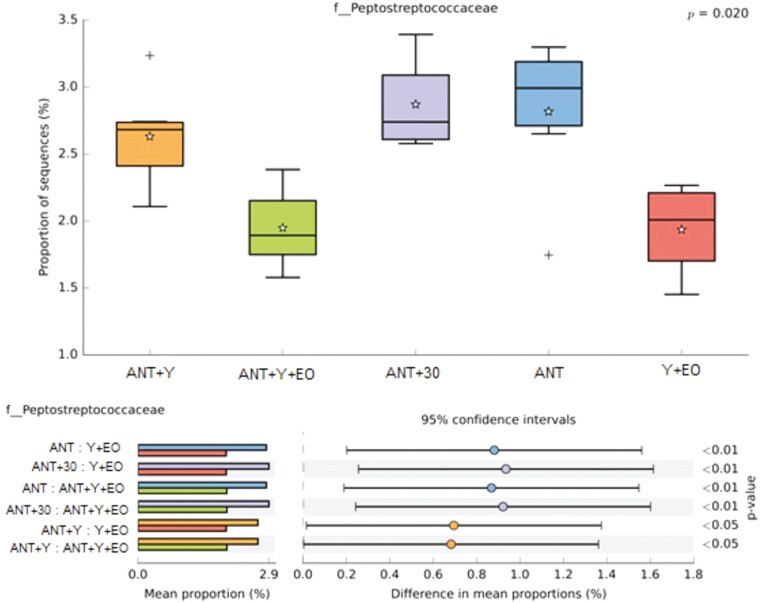
Effect of experimental diets on *Peptostreptococcaceae* family relative abundance in market hogs. (A) Box plot showing the distribution in the proportion of *Peptostreptococcaceae* assigned to samples from treatments. (B) Post hoc plot for *Peptostreptococcaceae* indicating. ANT+Y: basal diet with antibiotics and yeast products; ANT+Y+EO: basal diet with antibiotics, yeast products, and essential oils; ANT+30: basal diet with antibiotics in overdose; ANT: basal diet with antibiotics; Y+EO: basal diet with yeast products and essential oils. Piglet is an experimental unit; six piglets/treatment.

**Figure 7.**
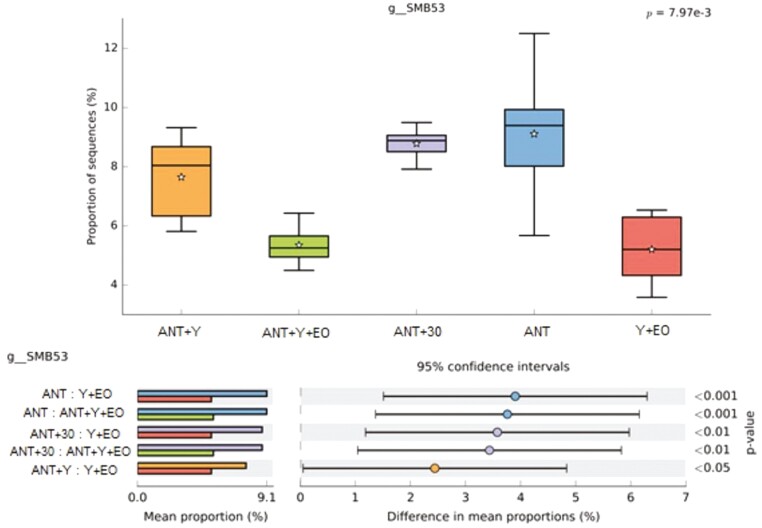
Effect of experimental diets on *SMB53* genus (*Clostridiaceae* family) relative abundance in market hogs. (A) Box plot showing the distribution in the proportion of *SMB53* genus assigned to samples from treatments. (B) Post hoc plot for *SMB53* genus indicating. ANT+Y: basal diet with antibiotics and yeast products; ANT+Y+EO: basal diet with antibiotics, yeast products and essential oils; ANT+30: basal diet with antibiotics in overdose; ANT: basal diet with antibiotics; Y+EO: basal diet with yeast products and essential oils. Piglet is an experimental unit; six piglets/treatment.

**Figure 8.**
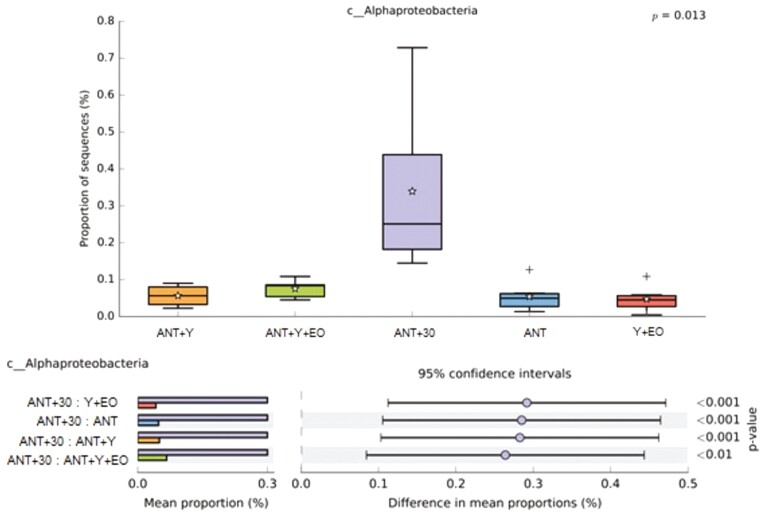
Effect of experimental diets on *Alphaproteobacteria* class relative abundance in market hogs. (A) Box plot showing the distribution in the proportion of *Alphaproteobacteria* genus assigned to samples from treatments. (B) Post hoc plot for *Alphaproteobacteria* genus indicating. ANT+Y: basal diet with antibiotics and yeast products; ANT+Y+EO: basal diet with antibiotics, yeast products, and essential oils; ANT+30: basal diet with antibiotics in overdose; ANT: basal diet with antibiotics; Y+EO: basal diet with yeast products and essential oils. Piglet is an experimental unit; six piglets/treatment.

Regarding the biodiversity indicators of the bacterial communities, alpha diversity by Shannon Entropy were lower in the ANT, ANT+30, and Y+EO groups than observed for the ANT+Y+EO group (*P* < 0.050), but similar between other groups (Fig. 2). The most abundant phyla on fecal samples were *Firmicutes*, *Bacteroidetes*, *Proteobacteria*, *Actinobacteria*, and *Spirochaetes* (Fig. 3)*. Firmicutes* phylum and F/B ratio were higher abundant in the ANT group than in the ANT+Y+EO group (*P* < 0.050, Fig. 4). *Proteobacteria* phylum relative abundance in ANT+Y+EO was similar to ANT+30 and higher than ANT, ANT+Y, and Y+EO (*P* < 0.050, Fig. 5). At lower taxonomic levels representing *Firmicutes* phyla, *Peptostreptococcaceae* family (a member of *Clostridia* Class) relative abundance was higher in ANT+30, ANT, and ANT+Y groups than in the ANT+Y+EO and Y+EO groups (*P* < 0.050, Fig. 6). ANT+Y+EO and Y+EO groups show a lower abundance (%) of *SMB53 g*enus (*Clostridiaceae* family) than ANT and ANT+30 groups. Y+EO had shown the lowest levels between all groups (*P* < 0.010; Fig. 7). *Alphaproteobacteria* class abundance was higher in the ANT+30 group than any other group (*P* < 0.050. Fig. 8).

## DISCUSSION

In-feed additives are used as long as they fulfill a variety of purposes. It is important that they are resistant to the processes used in in-feed manufacturing. In addition, they must be safe for animals and must not affect food safety. Those products should also share a positive image toward the public, who are increasingly sensitive to the way livestock production works (Gallois et al., 2009). Finally, these alternatives must be effective in their purpose, act as growth promoters and provide health benefits to pig. However, it is important to note that there is no single alternative to substitute in-feed antibiotics, and a combination of different alternatives to antibiotics may be the most promising method to reduce or replace antibiotics in animal feeds (Xu et al., 2021).

In this study, in the first two phases of evaluation, the replacement of antibiotics by additives maintained the BW and ADG of the pigs. In addition, the use of overdose promoted a greater BW than the use of additives alone. In the entire period, the total substitution of antibiotics by additives decreased the growth performance of pigs, but the reduction in PA use as well as the replacement of AGP by YP and EO additives did not interfere with the growth performance of pigs. The data regarding the consumption of antibiotics per treatment, followed as expected, so that the experimental groups with the replacement and reduction of the use of antimicrobials by the additives generated a lower intake of the three drugs used. This factor impacted the other variables. In the nursery phase, it is common to observe the replacement of antibiotics by additives without impairing growth performance (Xiong et al., 2015; Kiarie et al., 2018; Soler et al., 2018; Resende et al., 2020). This is mainly due to the improvement in intestinal morphology or digestibility (Long et al., 2018). Modulation of the microbiota is also widely observed (Resende et al., 2020). The changes are associated with the great challenges that weaning provides, as it is associated with intestinal inflammation and digestive disorders (Smith et al., 2010; Campbell et al., 2013; Moeser et al., 2017). In the growing-finishing phase, less results are found and can be inconsistent (Wierup, 2001). Cheng et al. (2018) confirmed the hypothesis that the use of EO as an alternative to antibiotics can improve intestinal epithelial absorptive function, gut microbiota composition, and antioxidative stress capacity in growing-finishing pigs, as well as Yan et al. (2011) when using an herb extract mixture. Xu et al. (2021) showed, through a meta-analysis, that plant extracts improve ADG and FCR for growing pigs and can be used as antibiotics substitute. The antimicrobial and anti-inflammatory properties of these additives change the microbiota and regulates intestinal permeability contributing to their antidiarrheal properties (Wang et al., 2017).

We observed a lower diarrhea incidence when EO were used in the protocol associated with antibiotics and YP, which contributes to these findings. However, some studies that evaluate the inclusion of additives, such as EO, plant extracts, or YP, in antibiotics-free diets, have not observed an effect on the growth performance of pigs (Davis et al., 2002; Janz et al., 2007; Yan et al., 2010; Lowell et al., 2018). This inconsistency may be attributed to the different additives levels and animals used in each study (Yan et al., 2012) or differences of optimum concentration, purity, molecular weight, conformation, chemical modification, and solubility of YP in the diet formulation (Luo et al., 2019). Moreover, in this phase, the pigs may have a more developed digestive system, improved immunity, and increased resistance to intestinal disorders as the pig become older (Yan et al., 2010).

The pigs from ANT and ANT+30 groups did not show differences in growth performance in any of the phases, even though there was a difference of 22.83% for the total consumption of antibiotics between treatments, which shows that overdose antibiotics can often be ineffective. Resistance to antibiotics is an inherent side effect associated with the overuse, abuse, or substantial use of ANT (Williams-Nguyen et al., 2016; Manyi-Loh et al., 2018; Albernaz-Gonçalves et al., 2021). When resistant strains are extracted from their host, they are disseminated into the environment, and increase the spread of resistant genes among bacterial populations (Lin et al., 2015). The diverse range of novel antibiotic-resistance genes could be accessible to clinically relevant bacteria and play a critical role in the emergence of antibiotic resistance among pathogens (Pehrsson et al., 2013). Besides being ineffective, overdose antibiotics negatively affect the alpha diversity, reducing the total genus content, compared with the replacement of AGP and reduction of antibiotics. Elimination of antibiotics in diet shows a similar effect. Reduced alpha diversity is related to a less mature and stable microbiota, with a reduction of functional redundancy (Chen et al., 2017; Trevisi et al., 2021). The loss of microbial diversity is referred to as gut dysbiosis (Wilkins et al., 2019) and the use of antibiotics is associated with intestinal microbiota imbalance (Stecher et al., 2013; Jo et al., 2021) and reduced diversity (Knecht et al., 2014). Just like there are still persistent long-term impacts on the intestinal microbiota that remain post-treatment (Cecilia et al., 2007; Yin et al., 2015), our studies showed that antibiotics overuse also strongly impacts the diversity.

The supply of the YP, for pigs that received antibiotics during the growing-finishing phase, did not modify the alpha diversity indicators, differently of the use of YP and EO. Changes in alpha diversity were also not observed by Xu et al. (2018) when supplementing weaned piglets with yeast probiotics. The effect of YP on the immune system has been investigated in many studies (Broadway et al., 2015), but the results in the enteric microbiota can be contradictory. In addition, an overall increasing trend in alpha community diversity of the gut microbiome is observed during aging (Wang et al., 2019), suggesting that the swine gut microbiome matures after the finishing phase, and this can contribute to the equivalence between the treatments that included the YP or not. Zhang et al. (2020) and Li et al. (2018) also found no differences in alpha diversity in their studies with EO for weaned piglets. As the increase in microbial diversity in this study was found with the addition of both additives, we can observe a synergism between them, favoring the intestinal health of these pigs. Microbiome diversity is used as a good indicator of gut health in humans (Menni et al., 2017) and animals (Hildebrand et al., 2013).

The most abundant phyla in fecal samples were *Firmicutes* and *Bacteroidetes*. The F/B ratio was highly abundant in ANT group than in ANT+Y+EO group. It is usual that the F/B ratio in swine gut microbiota gradually increases over time (Kim et al., 2012, 2015). Thus, ANT-treated pigs showed an accelerated increase in the F/B ratio (Kim et al., 2016). Pigs from ANT group received antibiotics the entire experimental period, unlike animals from the ANT+Y+EO group, promoting a 34.33% reduction in the total consumption of antibiotics by animals. A lower abundance of *Firmicutes* levels reflects on lower F/B ratios indicating a microbiome that favors gram-negative bacteria such as *Bacteroides, Alistipes, Parabacteroides*, and *Prevotella* (Stojanov et al., 2020).

*Peptostreptococcaceae f*amily (*Firmicutes* phylum) relative abundance was also higher when the antibiotics were used. The abundance of species of four families of the *Firmicutes* phylum (*Streptococcaceae*, *Peptococcaceae*, *Peptostreptococcaceae*, and *Clostridiaceae*) correlated positively with host weight gain (Kim et al., 2016). Firmicutes species metabolize available energy sources more effectively than *Bacteroidetes* species and consequently promote weight gain (Kallus and Brandt, 2012). Thus, manipulating gut microbial communities could control fat storage in pigs (Guo et al., 2008) and affect growth traits in pigs through host–microbe interactions (Lu et al., 2018; Oh et al., 2020).

ANT+Y+EO and Y+EO groups showed a lower abundance of *Clostridium*-related *SMB53* genera than ANT and ANT+30 groups. Pigs from Y+EO group, which did not receive antibiotics at any phase, had shown the lowest levels among all groups. Gao et al. (2018) found functional analysis during the weaning and nursery periods that showed a convergent presence of carbohydrate metabolism, amino acid metabolism, DNA replication and repair, and membrane transportation and the genera. The *SMB53* genus belongs to the *Clostridiaceae* family. Most members of this family have the ability to consume mucus- and plant-derived saccharides, such as glucose (Wuest et al., 2011). These findings are interesting because the pigs from Y+EO group showed lower growth performance during the evaluation, which may have happened due to poor use of feed ingredients. The fecal microbiota in high-feed efficiency pigs have a greater capacity to degrade dietary cellulose, polysaccharide, and protein and may have a greater abundance of microbes to promote intestinal health (Quan et al., 2019). In this study, the use of ANT seems to favor the microbiota for this purpose.

Greater FE increases profitability while reducing the environmental impact of pig production (Quan et al., 2020). This is especially important given that pig is one of the major sources of animal protein in human diet. The reduction in the use of antibiotics proposed in our study, and its effects on growth performance, fulfilled these assumptions and becomes a viable option. Furthermore, total withdrawal can be assessed as a possible protocol when requested, without causing major losses in productivity. In recent years, analyzing the pig microbiota has gained interest because it allows for the prediction of the functional and metabolic capacity of such communities, which are believed to impact all aspects of host physiology including nutrient processing, energy harvesting, and growth performance (Tan et al., 2017; Quan et al., 2019). Thus, a better understanding of how the additives interfere with the finishing pig’s microbiota is fundamental for the studies in the future since preventive protocols without antibiotics will be more and more demanded.

## CONCLUSIONS

In conclusion, our results indicate that the use of YP and EO as growth promoters and as prophylactic, in partial replacement to the in-feed antibiotic protocols, does not affect the growth performance, can replace antibiotic growth promotors, and reduces the use of PA in-feed in growing-finishing pigs. In addition, the use of YP and EO increased the microbial diversity, despite having important genera for weight gain in less abundance. Overdose of PA does not improve growth performance and reduces microbial diversity, which does not characterize it as an efficient preventive protocol.

## Supplementary Material

txae104_suppl_Supplementary_Table_S1

## References

[CIT0001] Agazzi, A., V. Perricone, F. Omodei Zorini, S. Sandrini, E. Mariani, X. -R. Jiang, A. Ferrari, M. Crestani, T. X. Nguyen, V. Bontempo, et al. 2020. Dietary mannan oligosaccharides modulate gut inflammatory response and improve duodenal villi height in post-weaning piglets improving feed efficiency. Animals (Basel) 10:1283. doi:10.3390/ani10081283 32731342 PMC7459834

[CIT0002] Albernaz-Gonçalves, R., G. Olmos, and M. J. Hötzel. 2021. Exploring farmers’ reasons for antibiotic use and misuse in pig farms in Brazil. Antibiotics (Basel) 10:331. doi:10.3390/antibiotics10030331 33809885 PMC8004152

[CIT0003] Backhed, F., J. Roswall, Y. Peng, Q. Feng, H. Jia, P. Kovatcheva-Datchary, Y. Li, Y. Xia, H. Xie, H. Zhong, et al. 2015. Dynamics and stabilization of the human gut microbiome during the first year of life. Cell Host Microbe 17:690–703. doi:10.1016/j.chom.2015.04.004 25974306

[CIT0004] Brenes, A., and E. Roura. 2010. Essential oils in poultry nutrition: main effects and modes of action. Anim. Feed Sci. Technol. 158:1–14. doi:10.1016/j.anifeedsci.2010.03.007

[CIT0005] Broadway, P. R., J. A. Carroll, and N. C. B. Sanchez. 2015. Live yeast and yeast cell wall supplements enhance immune function and performance in food-producing livestock: a review. Microorganisms 3:417–427. doi:10.3390/microorganisms3030417 27682097 PMC5023245

[CIT0006] Burdick Sanchez, N. C., P. R. Broadway, and J. A. Carroll. 2021. Influence of yeast products on modulating metabolism and immunity in cattle and swine. Animals (Basel) 11:371. doi:10.3390/ani11020371 33540746 PMC7913008

[CIT0007] Campbell, J. M., J. D. Crenshaw, and J. Polo. 2013. The biological stress of early weaned piglets. J. Anim. Sci. Biotechnol. 4:19. doi:10.1186/2049-1891-4-19 23631414 PMC3651348

[CIT0008] Caporaso, J. G., J. Kuczynski, J. Stombaugh, K. Bittinger, F. D. Bushman, E. K. Costello, N. Fierer, A. G. Peña, J. K. Goodrich, J. I. Gordon, et al. 2010. QIIME allows analysis of high-throughput community sequencing data. Nat. Methods 7:335–336. doi:10.1038/nmeth.f.303 20383131 PMC3156573

[CIT0009] Casey, P. G., G. E. Gardiner, G. Casey, B. Bradshaw, P. G. Lawlor, P. B. Lynch, F. C. Leonard, C. Stanton, R. P. Ross, G. F. Fitzgerald, et al. 2007. A five-strain probiotic combination reduces pathogen shedding and alleviates disease signs in pigs challenged with *Salmonella enterica serovar Typhimurium*. Appl. Environ. Microbiol. 73:1858–1863. doi:10.1128/AEM.01840-06 17261517 PMC1828830

[CIT0010] Cecilia, J., L. Sonja, E. Charlotta, and K. J. Janet. 2007. Long-term ecological impacts of antibiotic administration on the human intestinal microbiota. ISME J. 1:56–66. doi:10.1038/ismej.2007.3 18043614

[CIT0011] Chen, L., Y. Xu, X. Chen, C. Fang, L. Zhao, and F. Chen. 2017. The maturing development of gut microbiota in commercial piglets during the weaning transition. Front. Microbiol. 8:1–13. doi:10.3389/fmicb.2017.01688 28928724 PMC5591375

[CIT0012] Cheng, C., M. Xia, X. Zhang, C. Wang, S. Jiang, and J. Peng. 2018. Supplementing oregano essential oil in a reduced-protein diet improves growth performance and nutrient digestibility by modulating intestinal bacteria, intestinal morphology, and antioxidative capacity of growing-finishing pigs. Animals (Basel) 8:159. doi:10.3390/ani8090159 30235792 PMC6162377

[CIT0013] Chitprasert, P., and P. Sutaphanit. 2014. Holy basil (*Ocimum sanctum* Linn.) essential oil delivery to swine gastrointestinal tract using gelatin microcapsules coated with aluminum carboxymethyl cellulose and beeswax. J. Agric. Food Chem. 62:12641–12648. doi:10.1021/jf5019438 25382222

[CIT0014] Davis, M. E., C. V. Maxwell, D. C. Brown, B. Z. de Rodas, Z. B. Johnson, E. B. Kegley, D. H. Hellwig, and R. A. Dvorak. 2002. Effect of dietary mannan oligosaccharides and(or) pharmacological additions of copper sulfate on growth performance and immunocompetence of weanling and growing/finishing pigs. J. Anim. Sci. 80:2887–2894. doi:10.2527/2002.80112887x 12462256

[CIT0016] Degnan, P. H., and H. Ochman. 2012. Illumina-based analysis of microbial community diversity. ISME J. 6:183–194. doi:10.1038/ismej.2011.74 21677692 PMC3246231

[CIT0015] Dewulf, J., P. Joosten, I. Chantziaras, E. Bernaerdt, W. Vanderhaeghen, M. Postma, and D. Maes. 2022. Antibiotic use in European pig production: less is more. Antibiotics (Basel) 11:1493. doi:10.3390/antibiotics11111493 36358148 PMC9686698

[CIT0017] Gallois, M., H. J. Rothkötter, M. Bailey, C. R. Stokes, and I. P. Oswald. 2009. Natural alternatives to in-feed antibiotics in pig production: can immunomodulators play a role? Animal 3:1644–1661. doi:10.1017/S1751731109004236 22443549

[CIT0018] Gao, P., H. Wang, Z. Cheng, Y. Wang, P. Song, X. Guo, Z. Duan, G. Cao, J. Liu, M. Du, et al. 2018. Characterization and comparison of the intestinal microbiota in Chinese Shanxi black pig during the weaning and nursery periods. PeerJ Prepr. 6:e26893v1. doi:10.7287/peerj.preprints.26893v1

[CIT0019] Garrett, W. S., J. I. Gordon, and L. H. Glimcher. 2010. Homeostasis and inflammation in the intestine. Cell 140:859–870. doi:10.1016/j.cell.2010.01.023 20303876 PMC2845719

[CIT0020] Gaskins, H. R., C. T. Collier, and D. B. Anderson. 2002. Antibiotics as growth promotants: mode of action. Anim. Biotechnol. 13:29–42. doi:10.1081/ABIO-120005768 12212942

[CIT0021] Gong, J., F. Yin, Y. Hou, and Y. Yin. 2013. Review: Chinese herbs as alternatives to antibiotics in feed for swine and poultry production: potential and challenges in application. Can. J. Anim. Sci. 94:223–241. doi:10.4141/cjas2013-144

[CIT0022] Guo, X., X. Xia, R. Tang, J. Zhou, H. Zhao, and K. Wang. 2008. Development of a real‐time PCR method for Firmicutes and Bacteroidetes in faeces and its application to quantify intestinal population of obese and lean pigs. Lett. Appl. Microbiol. 47:367–373. doi:10.1111/j.1472-765X.2008.02408.x 19146523

[CIT0023] Heo, J. M., F. O. Opapeju, J. R. Pluske, J. C. Kim, D. J. Hampson, and C. M. Nyachoti. 2013. Gastrointestinal health and function in weaned pigs: a review of feeding strategies to control post-weaning diarrhoea without using in-feed antimicrobial compounds. J. Anim. Physiol. Anim. Nutr. (Berl). 97:207–237. doi:10.1111/j.1439-0396.2012.01284.x 22416941

[CIT0024] Hildebrand, F., T. Nguyen, B. Brinkman, R. G. Yunta, B. Cauwe, P. Vandenabeele, A. Liston, and J. Raes. 2013. Inflammation associated enterotypes, host genotype, cage and inter-individual effects drive gut microbiota variation in common laboratory mice. Genome Biol. 14:R4. doi:10.1186/gb-2013-14-1-r4 23347395 PMC4053703

[CIT0025] Janz, J. A. M., P. C. H. Morel, B. H. P. Wilkinson, and R. W. Purchas. 2007. Preliminary investigation of the effects of low-level dietary inclusion of fragrant essential oils and oleoresins on pig performance and pork quality. Meat Sci. 75:350–355. doi:10.1016/j.meatsci.2006.06.027 22063669

[CIT0026] Jo, H. E., M. -S. Kwon, T. W. Whon, D. W. Kim, M. Yun, J. Lee, M. -Y. Shin, S. -H. Kim, and H. -J. Choi. 2021. Alteration of gut microbiota after antibiotic exposure in finishing swine. Front. Microbiol. 12:596002. doi:10.3389/fmicb.2021.596002 33643231 PMC7906994

[CIT0027] Kallus, S. J., and L. J. Brandt. 2012. The intestinal microbiota and obesity. J. Clin. Gastroenterol. 46:16–24. doi:10.1097/MCG.0b013e31823711fd 22064556

[CIT0028] Kiarie, E., C. Voth, D. Wey, C. Zhu, P. Vingerhoeds, S. Borucki, and E. J. Squires. 2018. Comparative efficacy of antibiotic growth promoter and benzoic acid on growth performance, nutrient utilization, and indices of gut health in nursery pigs fed corn–soybean meal diet. Can. J. Anim. Sci. 98:868–874. doi:10.1139/cjas-2018-0056

[CIT0029] Kil, D. Y., and H. H. Stein. 2010. Invited review: management and feeding strategies to ameliorate the impact of removing antibiotic growth promoters from diets fed to weanling pigs. Can. J. Anim. Sci. 90:447–460. doi:10.4141/cjas10028

[CIT0030] Kim, H. B., K. Borewicz, B. A. White, R. S. Singer, S. Sreevatsan, Z. J. Tu, and R. E. Isaacson. 2012. Microbial shifts in the swine distal gut in response to the treatment with antimicrobial growth promoter, tylosin. Proc. Natl. Acad. Sci. USA 109:15485–15490. doi:10.1073/pnas.1205147109 22955886 PMC3458334

[CIT0031] Kim, J., R. B. Guevarra, S. G. Nguyen, J. H. Lee, D. K. Jeong, and T. Unno. 2016. Effects of the antibiotics growth promoter tylosin on swine gut microbiota. J. Microbiol. Biotechnol. 26:876–882. doi:10.4014/jmb.1512.12004 26869601

[CIT0032] Kim, J., S. G. Nguyen, R. B. Guevarra, I. Lee, and T. Unno. 2015. Analysis of swine fecal microbiota at various growth stages. Arch. Microbiol. 197:753–759. doi:10.1007/s00203-015-1108-1 25832348

[CIT0033] Knecht, H., S. C. Neulinger, F. A. Heinsen, C. Knecht, A. Schilhabel, R. A. Schmitz, A. Zimmermann, V. M. dos Santos, M. Ferrer, P. C. Rosenstiel, et al. 2014. Effects of β-lactam antibiotics and fluoroquinolones on human gut microbiota in relation to *Clostridium difficile* associated diarrhea. PLoS One 9:e89417. doi:10.1371/journal.pone.0089417 24586762 PMC3938479

[CIT0034] Kogan, G., and A. Kocher. 2007. Role of yeast cell wall polysaccharides in pig nutrition and health protection. Livest Sci. 109:161–165. doi:10.1016/j.livsci.2007.01.134

[CIT0035] Landers, T. F., B. Cohen, T. E. Wittum, and E. L. Larson. 2012. A review of antibiotic use in food animals: perspective, policy, and potential. Public Health Rep. 127:4–22. doi:10.1177/003335491212700103 22298919 PMC3234384

[CIT0036] Li, Y., X. Fu, X. Ma, S. Geng, X. Jiang, Q. Huang, C. Hu, and X. Han. 2018. Intestinal microbiome-metabolome responses to essential oils in piglets. Front. Microbiol. 9:1988. doi:10.3389/fmicb.2018.01988 30210470 PMC6120982

[CIT0037] Lin, J., K. Nishino, M. C. Roberts, M. Tolmasky, R. I. Aminov, and L. Zhang. 2015. Mechanisms of antibiotic resistance. Front. Microbiol. 6:1–3. doi:10.3389/fmicb.2015.00034 25699027 PMC4318422

[CIT0038] Liu, Y., T. M. Che, D. Bravo, and J. E. Pettigew. 2012. Anti-inflammatory effects of several plant extracts on porcine alveolar macrophages in vitro. J. Anim. Sci. 90:2774–2783. doi:10.2527/jas.2011-4304 22328722

[CIT0039] Liu, Y., C. D. Espinosa, J. J. Abelilla, G. A. Casas, L. V. Lagos, S. A. Lee, W. B. Kwon, J. K. Mathai, D. M. D. L. Navarro, N. W. Jaworski, et al. 2018. Non-antibiotic feed additives in diets for pigs: a review. Anim Nutr. 4:113–125. doi:10.1016/j.aninu.2018.01.007 30140751 PMC6103469

[CIT0040] Liu, Y., M. Song, T. M. Che, J. A. S. Almeida, J. J. Lee, D. Bravo, C. W. Maddox, and J. E. Pettigrew. 2013. Dietary plant extracts alleviate diarrhea and alter immune responses of weaned pigs experimentally infected with a pathogenic *Escherichia coli*. J. Anim. Sci. 91:5294–5306. doi:10.2527/jas.2012-6194 24045466

[CIT0041] Long, S. F., Y. T. Xu, L. Pan, Q. Q. Wang, C. L. Wang, J. Y. Wu, Y. Y. Wu, Y. M. Han, C. H. Yun, and X. S. Piao. 2018. Mixed organic acids as antibiotic substitutes improve performance, serum immunity, intestinal morphology and microbiota for weaned piglets. Anim. Feed Sci. Technol. 235:23–32. doi:10.1016/j.anifeedsci.2017.08.018

[CIT0042] Lowell, J. E., B. M. Bohrer, K. B. Wilson, M. F. Overholt, B. N. Harsh, H. H. Stein, A. C. Dilger, and D. D. Boler. 2018. Growth performance, carcass quality, fresh belly characteristics, and commercial bacon slicing yields of growing-finishing pigs fed a subtherapeutic dose of an antibiotic, a natural antimicrobial, or not fed an antibiotic or antimicrobial. Meat Sci. 136:93–103. doi:10.1016/j.meatsci.2017.10.011 29107868

[CIT0043] Lu, D., F. Tiezzi, C. Schillebeeckx, N. P. McNulty, C. Schwab, C. Shull, and C. Maltecca. 2018. Host contributes to longitudinal diversity of fecal microbiota in swine selected for lean growth. Microbiome 6:4. doi:10.1186/s40168-017-0384-1 29301569 PMC5755158

[CIT0044] Luo, J., D. Zeng, L. Cheng, X. Mao, J. Yu, B. Yu, and D. Chen. 2019. Dietary β-glucan supplementation improves growth performance, carcass traits and meat quality of finishing pigs. Anim. Nutr. 5:380–385. doi:10.1016/j.aninu.2019.06.006 31890915 PMC6920398

[CIT0045] Lynch, S. V., and O. Pedersen. 2016. The human intestinal microbiome in health and disease. N. Engl. J. Med. 375:2369–2379. doi:10.1056/NEJMra1600266 27974040

[CIT0046] Manyi-Loh, C., S. Mamphweli, E. Meyer, and A. Okoh. 2018. Antibiotic use in agriculture and its consequential resistance in environmental sources: potential public health implications. Molecules 23:795. doi:10.3390/molecules23040795 29601469 PMC6017557

[CIT0047] McCormack, U. M., T. Curião, S. G. Buzoianu, M. L. Prieto, T. Ryan, P. Varley, F. Crispie, E. Magowan, B. U. Metzler-Zebeli, D. Berry, et al. 2017. Exploring a possible link between the intestinal microbiota and feed efficiency in pigs. Appl. Environ. Microbiol. 83:e00380–e00317. doi:10.1128/AEM.00380-17 28526795 PMC5514681

[CIT0048] Menni, C., M. A. Jackson, T. Pallister, C. J. Steves, T. D. Spector, and A. M. Valdes. 2017. Gut microbiome diversity and high-fibre intake are related to lower long-term weight gain. Int. J. Obes. (Lond). 41:1099–1105. doi:10.1038/ijo.2017.66 28286339 PMC5500185

[CIT0049] Miguel, J. C., S. L. Rodriguez-Zas, and J. E. Pettigrew. 2004. Efficacy of a mannan oligosaccharide (Bio-Mos®) for improving nursery pig performance. J. Swine Health Prod. 12:296–307.

[CIT0050] Moeser, A. J., C. S. Pohl, and M. Rajput. 2017. Weaning stress and gastrointestinal barrier development: implications for lifelong gut health in pigs. Anim. Nutr. 3:313–321. doi:10.1016/j.aninu.2017.06.003 29767141 PMC5941262

[CIT0051] Oh, J. K., J. P. Chae, E. A. B. Pajarillo, S. H. Kim, M. ‐J. Kwak, J. ‐S. Eun, S. W. Chee, K. ‐Y. Whang, S. ‐H. Kim, and D. ‐K. Kang. 2020. Association between the body weight of growing pigs and the functional capacity of their gut microbiota. Anim. Sci. J. 91:e13418. doi:10.1111/asj.13418 32648357

[CIT0052] Omonijo, F. A., L. Ni, J. Gong, Q. Wang, L. Lahaye, and C. Yang. 2018. Essential oils as alternatives to antibiotics in swine production. Anim. Nutr. 4:126–136. doi:10.1016/j.aninu.2017.09.001 30140752 PMC6104524

[CIT0053] Pehrsson, E. C., K. J. Forsberg, M. K. Gibson, S. Ahmadi, and G. Dantas. 2013. Novel resistance functions uncovered using functional metagenomic investigations of resistance reservoirs. Front. Microbiol. 4:1–11. doi:10.3389/fmicb.2013.00145 23760651 PMC3675766

[CIT0054] Postler, T. S., and S. Ghosh. 2017. Understanding the holobiont: how microbial metabolites affect human health and shape the immune system. Cell Metab. 26:110–130. doi:10.1016/j.cmet.2017.05.008 28625867 PMC5535818

[CIT0055] Quan, J., G. Cai, M. Yang, Z. Zeng, R. Ding, X. Wang, Z. Zhuang, S. Zhou, S. Li, H. Yang, et al. 2019. Exploring the fecal microbial composition and metagenomic functional capacities associated with feed efficiency in commercial DLY pigs. Front. Microbiol. 10:52. doi:10.3389/fmicb.2019.00052 30761104 PMC6361760

[CIT0056] Quan, J., Z. Wu, Y. Ye, L. Peng, J. Wu, D. Ruan, Y. Qiu, R. Ding, X. Wang, E. Zheng, et al. 2020. Metagenomic characterization of intestinal regions in pigs with contrasting feed efficiency. Front. Microbiol. 11:32. doi:10.3389/fmicb.2020.00032 32038603 PMC6989599

[CIT0057] Resende, M., R. F. Chaves, R. M. Garcia, J. A. Barbosa, A. S. Marques, L. R. Rezende, A. P. Peconick, C. A. P. Garbossa, D. Mesa, C. C. Silva, et al. 2020. Benzoic acid and essential oils modify the cecum microbiota composition in weaned piglets and improve growth performance in finishing pigs. Livest. Sci. 242:104311. doi:10.1016/j.livsci.2020.104311

[CIT0058] Rostagno, H.S., Albino, L.F.T., Hannas, M.I., Donzele, J.L., Sakomura, N.K., Perazzo, F.G., Saraiva, A., Teixeira, M.L., Rodrigues, P.B., Oliveira, R.F., Barreto, S.L.T., and Brito, C.O. 2017. Tabelas brasileiras para aves e suínos: Composição de alimentos e exigências nutricionais [in Portuguese]. 4th ed. Editora UFV, Viçosa, Brazil.

[CIT0059] Sharifi-Rad, J., A. Sureda, G. C. Tenore, M. Daglia, M. Sharifi-Rad, M. Valussi, R. Tundis, M. Sharifi-Rad, M. R. Loizzo, A. O. Ademiluyi, et al. 2017. Biological activities of essential oils: from plant chemoecology to traditional healing systems. Molecules 22:70. doi:10.3390/molecules22010070 28045446 PMC6155610

[CIT0060] Smith, F., J. E. Clark, B. L. Overman, C. C. Tozel, J. H. Huang, J. E. Rivier, A. T. Blisklager, and A. J. Moeser. 2010. Early weaning stress impairs development of mucosal barrier function in the porcine intestine. Am. J. Physiol. Gastrointest. Liver Physiol. 298:G352–G363. doi:10.1152/ajpgi.00081.2009 19926814 PMC2838512

[CIT0061] Soler, C., T. Goossens, A. Bermejo, L. Migura-García, A. Cusco, O. Francino, and L. Fraile. 2018. Digestive microbiota is different in pigs receiving antimicrobials or a feed additive during the nursery period. PLoS One 13:e0197353. doi:10.1371/journal.pone.0197353 29799833 PMC5969774

[CIT0062] Sommer, F., and F. Backhed. 2013. The gut microbiota–masters of host development and physiology. Nat. Rev. Microbiol. 11:227–238. doi:10.1038/nrmicro2974 23435359

[CIT0063] Song, M., Y. Fan, H. Su, J. Ye, F. Liu, X. Zhu, L. Wang, P. Gao, G. Shu, Z. Wang, et al. 2019. Effects of actigen, a second-generation mannan rich fraction, in antibiotics-free diets on growth performance, intestinal barrier functions and inflammation in weaned piglets. Livest. Sci. 229:4–12. doi:10.1016/j.livsci.2019.09.006

[CIT0064] Spring, P., C. Wenk, A. Connolly, and A. Kiers. 2015. A review of 733 published trials on Bio-Mos®, a mannan oligosaccharide, and Actigen®, a second generation mannose rich fraction, on farm and companion animals. J. Appl. Anim. Nutr. 3:e8. doi:10.1017/jan.2015.6

[CIT0065] Stecher, B., L. Maier, and W. D. Hardt. 2013. ‘Blooming’ in the gut: how dysbiosis might contribute to pathogen evolution. Nat. Rev. Microbiol. 11:277–284. doi:10.1038/nrmicro2989 23474681

[CIT0066] Stier, H., V. Ebbeskotte, and J. Gruenwald. 2014. Immune-modulatory effects of dietary yeast beta-1,3/1,6-D-glucan. Nutr. J. 13:38. doi:10.1186/1475-2891-13-38 24774968 PMC4012169

[CIT0067] Stojanov, S., A. Berlec, and B. Štrukelj. 2020. The influence of probiotics on the *firmicutes/bacteroidetes* ratio in the treatment of obesity and inflammatory bowel disease. Microorganisms 8:1715–1716. doi:10.3390/microorganisms8111715 33139627 PMC7692443

[CIT0068] Tan, Z., Y. Wang, T. Yang, H. Ao, S. Chen, K. Xing, F. Zhang, X. Zhao, J. Liu, and C. Wang. 2018. Differences in gut microbiota composition in finishing Landrace pigs with low and high feed conversion ratios. Antonie Van Leeuwenhoek 111:1673–1685. doi:10.1007/s10482-018-1057-1 29497869 PMC6097733

[CIT0069] Tan, Z., T. Yang, Y. Wang, K. Xing, F. Zhang, X. Zhao, H. Ao, S. Chen, J. Liu, and C. Wang. 2017. Metagenomic analysis of cecal microbiome identified microbiota and functional capacities associated with feed efficiency in landrace finishing pigs. Front. Microbiol. 8:1546. doi:10.3389/fmicb.2017.01546 28848539 PMC5554500

[CIT0070] Tang, K. L., N. P. Caffrey, D. B. Nobrega, S. C. Cork, P. E. Ronksley, H. W. Barkema, A. J. Polachek, H. Ganshorn, N. Sharma, J. D. Kellner, et al. 2017. Restricting the use of antibiotics in food-producing animals and its associations with antibiotic resistance in food-producing animals and human beings: a systematic review and meta-analysis. Lancet Planet Health 1:e316–e327. doi:10.1016/S2542-5196(17)30141-9 29387833 PMC5785333

[CIT0071] Trevisi, P., D. Luise, F. Correa, and P. Bosi. 2021. Timely control of gastrointestinal eubiosis: a strategic pillar of pig health. Microorganisms 9:313–324. doi:10.3390/microorganisms9020313 33546450 PMC7913656

[CIT0072] Valpotić, H., M. Samardžija, S. Terzić, S. Vince, M. Šperanda, G. Lacković, B. Habrun, N. Mas, D. Đuričić, P. Kočila, et al. 2016. Effect of mannan oligosaccharide supplementation on blood and intestinal immune cells, bacteria numbers and performance in weaned pigs. Acta Vet. Brno 85:267–276. doi:10.2754/avb201685030267

[CIT0073] Van Boeckel, T. P., C. Brower, M. Gilbert, B. T. Grenfell, S. A. Levin, T. P. Robinson, A. Teillanta, and R. Laxminarayan. 2015. Global trends in antimicrobial use in food animals. Proc. Natl. Acad. Sci. USA 112:5649–5654. doi:10.1073/pnas.1503141112 25792457 PMC4426470

[CIT0074] Vannucci, L., J. Krizan, P. Sima, D. Stakheev, F. Caja, L. Rajsiglova, V. Horak, and M. Saieh. 2013. Immunostimulatory properties and antitumor activities of glucans (review). Int. J. Oncol. 43:357–364. doi:10.3892/ijo.2013.1974 23739801 PMC3775562

[CIT0075] Wang, D. F., L. L. Zhou, H. L. Zhou, G. Y. Hou, X. Zhou, and W. Li. 2017. Effects of *Piper sarmentosum* extract on the growth performance, antioxidant capability and immune response in weaned piglets. J. Anim. Physiol. Anim. Nutr. 101:105–112. doi:10.1111/jpn.12517 27045971

[CIT0076] Wang, X., T. Tsai, F. Deng, X. Wei, J. Chai, J. Knapp, J. Apple, C. V. Maxwell, J. A. Lee, Y. Li, et al. 2019. Longitudinal investigation of the swine gut microbiome from birth to market reveals stage and growth performance associated bacteria. Microbiome 7:109. doi:10.1186/s40168-019-0721-7 31362781 PMC6664762

[CIT0077] Wierup, M. 2001. The Swedish experience of the 1986 year ban of antimicrobial growth promoters, with special reference to animal health, disease prevention, productivity, and usage of antimicrobials. Microb. Drug Resist. 7:183–190. doi:10.1089/10766290152045066 11442345

[CIT0078] Wilkins, L. J., M. Monga, and A. W. Miller. 2019. Defining dysbiosis for a cluster of chronic diseases. Sci. Rep. 9:12918. doi:10.1038/s41598-019-49452-y 31501492 PMC6733864

[CIT0079] Williams-Nguyen, J., J. B. Sallach, S. Bartelt-Hunt, A. B. Boxall, L. M. Durso, J. E. McLain, R. S. Singer, D. D. Snow, and J. L. Zilles. 2016. Antibiotics and antibiotic resistance in agroecosystems: state of the science. J. Environ. Qual. 45:394–406. doi:10.2134/jeq2015.07.0336 27065386

[CIT0080] Wuest, P. K., M. A. Horn, and H. L. Drake. 2011. *Clostridiaceae* and *Enterobacteriaceae* as active fermenters in earthworm gut content. ISME J. 5:92–106. doi:10.1038/ismej.2010.99 20613788 PMC3105676

[CIT0081] Xiao, Y., K. Li, Y. Xiang, W. Zhou, G. Gui, and H. Yang. 2017. The fecal microbiota composition of boar Duroc, Yorkshire, Landrace and Hampshire pigs. Asian Australas. J. Anim. Sci. 30:1456–1463. doi:10.5713/ajas.16.0746 28231697 PMC5582331

[CIT0082] Xiong, X., H. Yang, B. Li, G. Liu, R. Huang, F. Li, P. Liao, Y. Zhang, C. M. Nyachoti, and D. Deng. 2015. Dietary supplementation with yeast product improves intestinal function, and serum and ileal amino acid contents in weaned piglets. Livest. Sci. 171:20–27. doi:10.1016/j.livsci.2014.10.012

[CIT0083] Xu, B., J. Fu, L. Zhu, Z. Li, M. Jin, and Y. Wang. 2021. Overall assessment of antibiotic substitutes for pigs: a set of meta-analyses. J. Anim. Sci. Biotechnol. 12:1–15. doi:10.1186/s40104-020-00534-2 33413687 PMC7792336

[CIT0084] Xu, J., Y. Li, Z. Yang, C. Li, H. Liang, Z. Wu, and W. Pu. 2018. Yeast probiotics shape the gut microbiome and improve the health of early-weaned piglets. Front. Microbiol. 9:2011. doi:10.3389/fmicb.2018.02011 30210480 PMC6119770

[CIT0085] Yan, L., Q. W. Meng, and I. H. Kim. 2011. The effect of an herb extract mixture on growth performance, nutrient digestibility, blood characteristics and fecal noxious gas content in growing pigs. Livest. Sci. 141:143–147. doi:10.1016/j.livsci.2011.05.011

[CIT0086] Yan, L., Q. W. Meng, and I. H. Kim. 2012. Effects of fermented garlic powder supplementation on growth performance, nutrient digestibility, blood characteristics and meat quality in growing‐finishing pigs. Anim. Sci. J. 83:411–417. doi:10.1111/j.1740-0929.2011.00973.x 22574793

[CIT0087] Yan, L., J. P. Wang, H. J. Kim, Q. W. Meng, X. Ao, S. M. Hong, and I. H. Kim. 2010. Influence of essential oil supplementation and diets with different nutrient densities on growth performance, nutrient digestibility, blood characteristics, meat quality and fecal noxious gas content in grower–finisher pigs. Livest. Sci. 128:115–122. doi:10.1016/j.livsci.2009.11.008

[CIT0088] Yang, H., X. Huang, S. Fang, W. Xin, L. Huang, and C. Chen. 2016. Uncovering the composition of microbial community structure and metagenomics among three gut locations in pigs with distinct fatness. Sci. Rep. 6:27427. doi:10.1038/srep27427 27255518 PMC4891666

[CIT0089] Yilmaz, P., L. W. Parfrey, P. Yarza, J. Gerken, E. Pruesse, C. Quast, T. Schweer, J. Peplies, W. Ludwig, and F. O. Glöckner. 2014. The SILVA and “All-species Living Tree Project (LTP)” taxonomic frameworks. Nucleic Acids Res. 42:D643–D648. doi:10.1093/nar/gkt1209 24293649 PMC3965112

[CIT0090] Yin, J., M. Prabhakar, S. Wang, S. X. Liao, X. Peng, Y. He, Y. R. Chen, H. F. Shan, J. Su, Y. X. Jiang, et al. 2015. Different dynamic patterns of β-lactams, quinolones, glycopeptides and macrolides on mouse gut microbial diversity. PLoS One 10:e0126712. doi:10.1371/journal.pone.0126712 25970622 PMC4430517

[CIT0091] Zhang, W., Y. Zhang, X. Zhang, Z. Deng, J. Liu, M. He, and H. Wang. 2020. Effects of dietary supplementation with combination of tributyrin and essential oil on gut health and microbiota of weaned piglets. Animals (Basel) 10:180. doi:10.3390/ani10020180 31973120 PMC7070613

[CIT0092] Zhao, P. Y., J. H. Jung, and I. H. Kim. 2012. Effect of mannan oligosaccharides and fructan on growth performance, nutrient digestibility, blood profile, and diarrhea score in weanling pigs. J. Anim. Sci. 90:833–839. doi:10.2527/jas.2011-3921 21984718

[CIT0093] Zhaxi, Y., X. Meng, W. Wang, L. Wang, Z. He, X. Zhang, and W. Pu. 2020. Duan-nai-An, a yeast probiotic, improves intestinal mucosa integrity and immune function in weaned piglets. Sci. Rep. 10:4556. doi:10.1038/s41598-020-61279-6 32165666 PMC7067797

