# Supplementary material for: A new approach: preventive protocols with yeast products and essential oils can reduce the in-feed use of antibiotics in growing-finishing pigs
Source: Transl Anim Sci. 2024 Jul 13;8:txae104. doi: 10.1093/tas/txae104 (PMC11344245; doi:10.1093/tas/txae104)
Supplement: txae104_suppl_Supplementary_Table_S1 [file txae104_suppl_supplementary_table_s1.docx]

**Supplementary table S1.** Basal diet composition (as-fed basis).

| **Ingredients, %** | **Growing phase** | | | | **Finishing phase** | |
| --- | --- | --- | --- | --- | --- | --- |
|  | **Phase I** | **Phase II** | **Phase III** | **Phase IV** | **Phase I** | **Phase II** |
|  | **(0-14 days)** | **(14-35 days)** | **(35-49 days)** | **(49-70 days)** | **(70-84 days)** | **(84-105 days)** |
| Corn, 7.86% CP^1^ | 58.71 | 67.41 | 72.89 | 78.23 | 77.80 | 75.79 |
| Soybean meal, 46% CP | 33.70 | 26.60 | 21.40 | 16.90 | 17.00 | 19.80 |
| Soybean oil | 3.30 | 2.50 | 2.30 | 1.90 | 2.00 | 2.00 |
| Dicalcium phosphate 18.5% P^2^ | 1.80 | 1.45 | 1.25 | 1.10 | 1.10 | 1.00 |
| Limestone | 0.76 | 0.66 | 0.60 | 0.56 | 0.56 | 0.52 |
| Salt | 0.47 | 0.43 | 0.40 | 0.40 | 0.40 | 0.35 |
| L-Lysine HCl | 0.36 | 0.36 | 0.36 | 0.36 | 0.36 | 0.16 |
| DL-Methionine | 0.18 | 0.13 | 0.10 | 0.09 | 0.09 | 0.01 |
| L-Threonine | 0.15 | 0.13 | 0.12 | 0.11 | 0.11 | 0.01 |
| L-Tryptophan | 0.03 | 0.03 | 0.03 | 0.04 | 0.04 | 0.00 |
| Vitamins and microminerals* | 0.16 | 0.16 | 0.16 | 0.16 | 0.16 | 0.16 |
| Inert | 0.40 | 0.15 | 0.40 | 0.17 | 0.40 | 0.21 |
| **Calculated values** |  |  |  |  |  |  |
| ME, Kcal/kg | 3350.00 | 3350.00 | 3350.00 | 3350.00 | 3350.00 | 3350.00 |
| Crude protein, % | 20.45 | 17.86 | 15.91 | 14.29 | 14.29 | 15.09 |
| SID^4^ Lys, % | 1.23 | 1.07 | 0.95 | 0.84 | 0.84 | 0.75 |
| SID Met, % | 0.45 | 0.37 | 0.32 | 0.29 | 0.29 | 0.23 |
| SID Met + Cys, % | 0.74 | 0.63 | 0.56 | 0.51 | 0.51 | 0.46 |
| SID Thr, % | 0.80 | 0.69 | 0.61 | 0.55 | 0.55 | 0.49 |
| SID Val, % | 0.25 | 0.21 | 0.19 | 0.17 | 0.17 | 0.15 |
| SID Ile, % | 0.85 | 0.74 | 0.65 | 0.58 | 0.58 | 0.63 |
| Total calcium, % | 0.77 | 0.66 | 0.57 | 0.50 | 0.50 | 0.55 |
| Available phosphorus, % | 0.87 | 0.73 | 0.63 | 0.57 | 0.57 | 0.55 |

^*^Levels per kg of premix. Minerals: 80 g of iron, 10 g of manganese, 200 mg of cobalt, 116 g of zinc, 1228 mg of iodine, 350 mg of selenium, 15 g of cooper. Vitamins: 17,000,000 IU of vitamin A, 6,000,000 IU of vitamin D3, 70,000 IU of vitamin E, 6,000 mg of vitamin K3, 3,200 mg of vitamin B1, 8,500 mg of vitamin B2, 35,000 mg of vitamin pantothenic acid, 6,000 mg of vitamin B6, 44 mg of vitamin B12, 44,000 mg of nicotinic acid, 1,600 mg of folic acid, 240 mg of biotin, 600 g of choline.

^1^CP = Crude protein; ^2^P = phosphorus; ^3^SID = standardized ileal digestible.
